# Comparison of the Protective Effects of Nebivolol and Metoprolol against LPS-Induced Injury in H9c2 Cardiomyoblasts

**DOI:** 10.3390/cimb45110583

**Published:** 2023-11-20

**Authors:** Rukhsana Gul, Meshail Okla, Amer Mahmood, Shahid Nawaz, Amina Fallata, Arwa Bazighifan, Musaad Alfayez, Assim A. Alfadda

**Affiliations:** 1 Obesity Research Center, College of Medicine, King Saud University, P.O. Box 2925, Riyadh 11461, Saudi Arabia; shnawaz@ksu.edu.sa (S.N.); arwa.baz@hotmail.com (A.B.); aalfadda@ksu.edu.sa (A.A.A.); 2 Department of Community Health Sciences, College of Applied Medical Sciences, King Saud University, P.O. Box 22452, Riyadh 11495, Saudi Arabia; 3 Stem Cell Unit, Department of Anatomy, College of Medicine, King Saud University, P.O. Box 2925, Riyadh 11461, Saudi Arabia; 4 Department of Medicine, College of Medicine, King Saud University, P.O. Box 2925, Riyadh 11461, Saudi Arabia

**Keywords:** nebivolol, metoprolol, LPS, oxidative stress, mitochondrial dysfunction, beta-blockers

## Abstract

Here, we, for the first time, compared the cardioprotective effects of third-generation vasodilating beta-blocker nebivolol (Neb) and conventional beta-blocker metoprolol (Met) on LPS-induced injury in H9c2 cardiomyoblasts. Our findings denoted that Neb and Met pretreatment diminish LPS-mediated cytotoxicity and oxidative stress. Concomitantly, LPS-triggered inflammatory cytokines activation was significantly suppressed by Neb but not by Met. Pretreatment with either Neb or Met alleviated LPS-mediated mitochondrial impairment by enhancing the expression of genes related to its biogenesis such as PGC-1α, NRF1, and TFAM. On the contrary, Neb but not Met-upregulated mitochondrial fusion-related genes such as OPA, and MFN2. In summary, our findings suggest that Neb and Met treatment significantly ameliorated the LPS-induced cytotoxicity and oxidative stress. Additionally, these findings suggest that Neb but not Met significantly down-regulates LPS-induced proinflammatory factors, probably by enhancing mitochondrial biogenesis and fusion.

## 1. Introduction

Myocarditis is an inflammation of the cardiac muscle that may lead to non-ischemic cardiomyopathy and may predispose some patients for mechanical circulatory support or even heart transplantation [1]. Bacterial myocarditis is caused by bacterial infection or the detrimental effects of bacterial products such as Lipopolysaccharide (LPS) [2]. LPS is involved in multiple organ pathologies, particularly myocarditis, which is induced by the stimulation of pro-inflammatory cytokines. Beta-blockers are usually recommended in cases of acute myocarditis, especially in cases of heart failure or arrhythmias [3]. Among the beta-blockers, nebivolol (Neb) is a third-generation selective β1-AR antagonist with vasodilator properties, an approved drug for the treatment of hypertension [4]. Neb is also a beta-blocker of choice as an option to treat heart failure when used as an adjunct to second-generation beta-blockers such as metoprolol (Met) [5]. Studies have linked the cardioprotective effects of Neb to its antioxidant activity and its role in the prevention of oxidative damage owing to its ability to decrease superoxide generation by inhibiting NADPH-oxidase activation [4,6,7,8]. The nebivolol-mediated increase in the bioavailability of vasodilators such as nitric oxide improves vasodilation and decreases endothelial dysfunction [7].The vasodilator property of Neb is arbitrated by the activation of endothelial NO synthase (eNOS), which may result from induced β3 adrenergic receptor activation or the decreased circulating levels of eNOS inhibitor asymmetric dimethylarginine [9]. NOS inhibition by NG-nitro-L-arginine methyl ester (L-NAME) reduced the vasodilatory properties of Neb on the isolated pulmonary artery [10]. Furthermore, the beneficial effects of Neb on improving insulin resistance and preventing weight gain are probably linked with improved diastolic function in patients on Neb [6,11]. On the other hand, Met is a traditional, non-vasodilatory β-blocker that is widely used to improve heart function and pathological cardiac hypertrophy [12,13,14]. The cardioprotective effects of Met have been reported previously in rodent models with LPS-induced inflammation. Compared to other clinically approved β1-selective blockers such as atenolol, and propranolol, Met disrupts neutrophil dynamics during exacerbated inflammation induced by ischemia–reperfusion injury [15].

Previous studies have compared the effects of Neb to Met on oxidative stress and inflammation [16]. Both Neb and Met significantly reduced brachial blood pressure and the plasma levels of oxLDL in patients with mild-to-moderate essential hypertension after one year of treatment. Serg et al. reported that the long-term administration of both Neb and Met has anti-inflammatory effects, with Neb being more effective against oxidative stress, independent of its antihypertensive effect [17]. In pulmonary endothelial cells, Neb was more potent than Met in decreasing vasoactive and proinflammatory factor production and controlling cell proliferation. Neb was also more effective than Met in restoring cardiac functions and inflammatory response in animals with pulmonary hypertension [10]. Additionally, in a model of angiotensin II-induced oxidative stress, Neb but not Met improved endothelial functions, increased the bioavailability of NO, and decreased the activation and expression of the NADPH oxidase complex and consequent superoxide formation [10].

In early stages of sepsis, the over-activation of sympathetic adrenergic system can lead to an increase in tachycardia, and vasoconstriction, which, if left untreated, can exerbate clinical severity of the condition and ultimately result in a higher mortality rate [18]. Clinical trials on sepsis-related cardiomyopathy under β-blockade are limited. Nevertheless, a few studies have reported improvements in heart rate, cardia function, and mortality rate during septic shock [19]. Morelli et al., 2013, conducted a randomized controlled trial on patients with severe septic shock, where 77 of them were randomly assigned to receive a continuous infusion of the short-acting β-blocker esmolol titrated to maintain a heart rate between 80 and 94/min. They found that the use of esmolol in those patients was associated with a reduction in the heart rate to achieve target levels, without an increase in adverse outcomes compared to those who received a standard care [20]. Additionally, Morelli and coworkers (2016) reported a reduction in the heart rate and an improvement in the static arterial elastance in a prospective observational study conducted on 45 septic shock patients receiving a titrated infusion to maintain heart rate between 80 and 94/min [20]. Furthermore, in a small randomized pilot trial conducted by Wang et al., in 2015, the combination of esmolol and milrinone (phosphodiesterase III inhibitor) for the treatment of severe sepsis, yielded improved heart rate control and enhanced 28-day survival rates in when compared to the groups that received milrinone only and control groups [21].

Although, studies have separately reported the protective effects of both Neb and Met previously, a direct comparison of their protective effects against from LPS-induced injury and dysfunctions in cardiomyocytes has not been reported to date. Our data reveal that both Neb and Met offer protection against LPS-induced oxidative stress, improve the antioxidant defense system, decrease mitochondrial ROS generation, and increase mitochondrial biogenesis. Notably, Neb but not Met also exhibits protective effects against LPS-induced inflammatory signaling and the induction of mitochondrial fusion related genes.

## 2. Materials and Methods

### 2.1. H9c2 Cell Culture

H9c2 embryonic rat-heart-derived cells (myoblast) (ATCC, CRL-1446) were cultured at 37 °C, in a CO_2_-air incubator in DMEM (ATCC 30-2002) enriched with 10% FBS (ATCC, 30-2020) and antibiotics (penicillin/streptomycin 100 U/mL: 100 mg/mL). Neb, Met, and LPS were purchased from Sigma-Aldrich (St. Louis, MO, USA) as pure reagent grade unless otherwise specified.

### 2.2. Cell Viability Assay

H9c2 cells plated in a 96-well plate were treated with Neb and Met in DMEM culture media for 24 h. The cells were then stimulated with LPS for 48 h. Subsequently, 20 µL/well of Cell Titer-Blue^®^ Reagent (Cell Titer-Blue (R) Cell Viability Assay, Promega, Madison, WI, USA) was added for 4 h. Cell viability was measured as per the manufacturer’s protocol. The viability of cultured cells was also assessed using a crystal violet dye. Treated and untreated cells in 96-well plates were stained with 50 µL of 0.5% crystal violet staining solution for 20 min on a rocking shaker. The cells were air-dried for 2 h and fixed with 200 µL of methanol for 20 min. The optical density of stained cells was measured at 570 nm with a microplate reader [22]. 

### 2.3. RNA Isolation and Quantitative Polymerase Chain Reaction (qPCR)

TRIzol reagent (Invitrogen, Carlsbad, CA, USA) was used to extract the total RNA from H9c2 cells according to the manufacturer’s protocol. RNA quantity was determined using nanodrop (Thermo Scientific™ NanoDrop 2000c, Waltham, MA, USA). Reverse transcription to synthesize cDNA was performed using reverse transcription kits (Omniscript RT Kit, Qiagen, DE, Hilden, Germany) with a total volume of 20 μL, using 400 ng of total RNA. qPCR was performed using SYBR Green mix (Applied Biosystems, Waltham, MA, USA) in the 7500 Real-Time PCR system (Applied Biosystems, USA). Expression of the target gene was normalized to house-keeping gene β-actin, which served as an endogenous control gene. The relative mRNA expression level was quantified using a comparative cycle threshold (CT) (2^−ΔΔCT^) [23]. Primers Table 1 were obtained from Macrogen Inc., (Seoul, Republic of Korea). 

### 2.4. Detection of Intracellular ROS

The dye 2′, 7′dichlorofluoresceinn diacetate (H2DCFDA; Molecular Probe, Eugene, OR, USA) was used for ROS detection. This cell-permeant dye is oxidized in the presence of ROS to yield a fluorescence molecule 2′, 7′-dichlorofluorescein (DCF) as described previously [23]. Cells plated in 96-well plates were incubated with H2DCFDA (4 μM) for 30 min in 5% CO_2_-air and maintained at 37 °C. Cells were rinsed twice with DPBS and pretreated with Neb and Met for 30 min, followed by LPS. Following LPS stimulation, ROS generation was measured for 2 h by measuring the fluorescence with (excitation = 488 nm and emission = 520 nm, respectively) using a microplate reader: Synergy HT multi-mode reader (BioTek Instruments, Inc., Winooski, VT, USA). 

### 2.5. Western Blotting

After cell lysis, Bradford protein assay (BCA Protein Assay, Pierce, Waltham, MA, USA) was used to determine the protein concentration of the lysates. Samples containing equal amounts of proteins (30–50 μg/lane) were separated by 10 or 12% SDS-PAGE and transferred to polyvinylidene difluoride (PVDF) membranes (Bio-Rad Laboratories, Hercules, CA, USA). Membranes were then blocked with 3% bovine serum albumin (BSA) (Sigma, St. Louis, MO, USA) for 1 h at room temperature, and then incubated with primary antibodies (1:1000 dilution) for gp91phox (NOX2) (Santa Cruz Biotechnology, Dallas, TX, USA), TNFα (Invitrogen, Carlsbad, CA, USA), NFkB (Cell Signaling Technology Inc., Boston, MA, USA) and iNOS (Cell Signaling Technology Inc., Boston, MA, USA) overnight at 4 °C. After washing 3 times with 1× Tris-buffered saline (TBS)–Tween-20 (TBS-T) buffer, membranes were incubated with (1:2000) secondary antibodies (peroxidase-conjugated IgG) for 2 h at room temperature. Following secondary antibody incubation, membranes were again washed 3 times with 1× TBS-T. For imaging, the membranes were briefly incubated with a chemiluminescence solution, and images were taken by gel documentation system G: BOX (Syngene, Cambridge, UK). For quantification of bands image analysis GeneTools software 4.0 (Syngene, Cambridge, UK) Quantitation of protein band density was carried out using GeneTools image analysis software (Syngene, Cambridge, UK), and target protein band density was normalized to the density of β-actin.

### 2.6. Detection of Mitochondrial ROS

MitoSOX™Red (Invitrogen, Carlsbad, CA, USA) was used for the detection of super-oxide in the mitochondria. The treated cells and control, in a 12-well plate, were incubated with MitoSOX Red (1.5 μM) at 37 °C. The cells were co-incubated with MitoTracker^®^ Green FM (Invitrogen, Carlsbad, CA, USA), (100 nM) in order to determine the mitochondrial content. After incubation, the staining solution was replaced with fresh, prewarmed serum free-media (SFM). The images were obtained by a fluorescence imager (Floid Cell Imaging Solution; Thermo Fisher Scientific, Waltham, MA, USA) and fluorescence intensities were measured by using Image J software 1.5. The mitochondrial ROS production is expressed as fold change over an untreated control group.

### 2.7. Statistical Analysis

Data are presented as mean ± standard error of the mean (SEM). Normality of data was determined for each group by a Shapiro–Wilk test. The alterations in the different groups were compared using one-way analysis of variance (ANOVA) followed by Scheffe’s *t*-test. Two-group analysis was conducted by Student’s *t*-test. A *p*-value of < 0.05 was considered to be statistically significant.

## 3. Results

### 3.1. Effects of Neb and Met on LPS-Mediated Cellular and Mitochondrial ROS Generation in H9c2 Cardiomyoblasts

**Figure 1:**
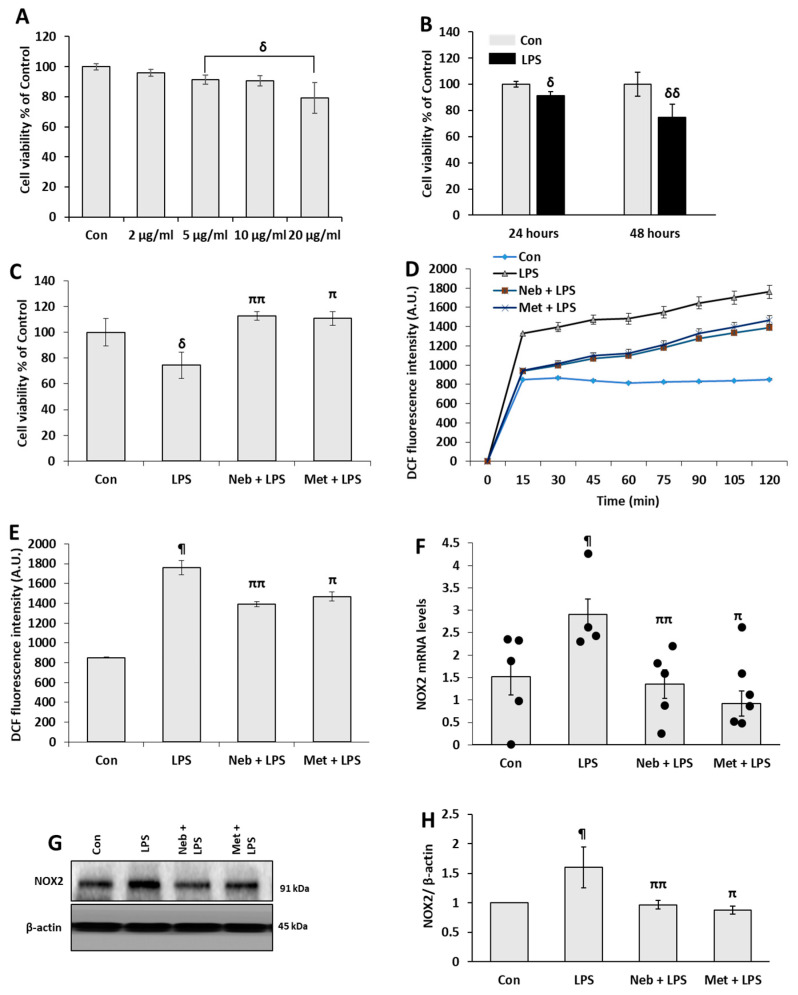
**Neb and Met reduced LPS-induced cytotoxicity and ROS generation in H9c2 cells:** (**A**). The cytotoxicity effect of increasing LPS concentrations on H9c2 using cell titer assay. (**B**). The cytotoxic effect of 5 µg/mL LPS for 24 and 48 h obtained by cell titer assay. (**C**). Cell viability results were obtained by cell titer assay following treatment with Neb 1 µM, and Met 10 µM in the presence or absence of LPS. (**D**,**E**) ROS generation was evaluated by DCFDA fluorescence. LPS treatment raised DCF fluorescence, which was markedly reduced by neb and met pretreatment. (**F**) mRNA levels of NOX2 were downregulated by pretreatment with both Neb and Met. (**G**,**H**) The immunoblot and corresponding histogram illustrate the protein expression of NOX2, β-actin was used as an internal control. ^δ^ *p* < 0.02, ^δδ^ *p* < 0.01, ^¶^ *p* < 0.05 vs. untreated (Con), ^ππ^ *p* < 0.02, ^π^ *p* < 0.05 vs. LPS. Values are presented as means ± SEM N ≥ 6 for each treatment group.

First, LPS cytotoxicity against H9c2 cells was evaluated by MTT assay. Cells were subjected to increasing concentrations of LPS (0, 2, 5, 10 or 20 µg/mL) for 24 h. We found that cell viability was affected by LPS in a dose-dependent manner (Figure 1A). A dose of 5 µg/mL of LPS for 48 h decreases cell viability by 75% (Figure 1B), thus, this concentration and time point were used for all subsequent experiments. Then we investigated the cytotoxic effects of LPS in the presence and absence of Neb and Met in H9c2 cells. Cells were treated with Neb (1 µm), and Met (10 µm) before stimulation with LPS (5 µg/mL) for 48 h. The cell titer assay results demonstrated a significant reduction in cell viability by LPS in comparison to the control group. However, pretreatment with Neb and Met reduced the cytotoxic effects of LPS and significantly increased the cell viability of H9c2 myocardial cells (Figure 1C). For the effect of Neb and Met on cell viability, we reconfirmed our results using crystal violet assay. Our data are consistent with the above results (Supplementary Figure S1).

Next, we evaluated Neb and Met’s effects on LPS-induced oxidative stress in H9c2 cells, cells were pretreated with Neb and Met for 30 min prior to stimulation with LPS (5 µg/mL). As shown in Figure 1D, cells treated with LPS showed an increase in intracellular ROS generation in comparison to control, as shown by DCF fluorescence intensity. Pretreatment with Neb and Met significantly reduced DCF fluorescence intensity in comparison to LPS treatment (Figure 1D,E). We also looked at the effects of Neb and Met effects on LPS-induced NOX2 (catalytic subunit of NADPH oxidase) mRNA (Figure 1F) and protein (gp91phox) expression (Figure 1G,H). Consistent with our above findings, nebivolol, and Met pretreatment leads to a reduction in both mRNA and protein expression levels of NOX2 following LPS treatment. 

### 3.2. Effects of Neb and Met on LPS-Stimulated Gene and Protein Expression Levels of Inflammatory Markers in H9c2 Cardiomyoblasts

**Figure 2:**
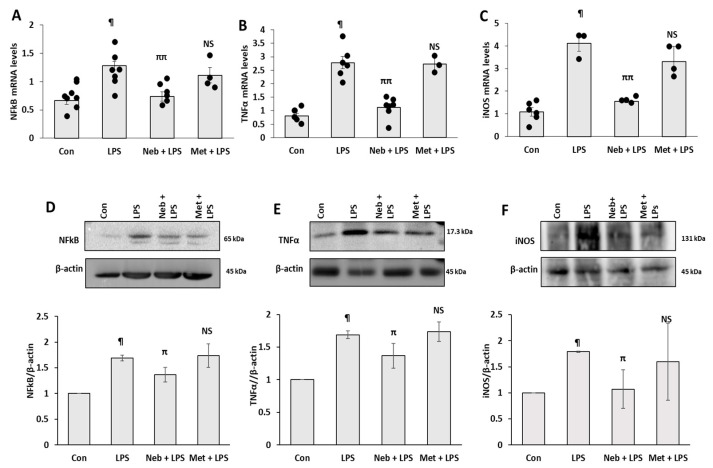
**Neb but not Met reduced the gene and protein expressions of inflammatory markers in LPS-stimulated H9c2 cells:** Downregulation of mRNA expression levels of NF-κB (**A**), TNF-α (**B**) and iNOS (**C**) by Neb but not Met. (**D**–**F**). The immunoblots and corresponding histograms illustrate the expression of NFkB, TNFα and iNOS respectively. β-actin was used as an internal control. ^¶^ *p* < 0.05 vs. Con, ^ππ^ *p* < 0.02, ^π^ *p* < 0.05 vs. LPS. and NS nonsignificant vs. LPS. Values are presented as means ± SEM N ≥ 6 for each treatment group.

To examine the effects of Neb and Met on the inflammation triggered by LPS, the expressions of inflammatory factors were evaluated. Exposure to LPS alone significantly elevated expressions of inflammatory genes such as NF-kB, TNF-α and iNOS compared to untreated control in H9c2 cells. As shown in Figure 2A–C, pretreatment with Neb dramatically suppressed LPS-stimulated increases in gene expression levels of NF-kB, TNF-α, and iNOS. However, in contrast to the above findings treatment with Met did not alter the LPS-stimulated increases in the mRNA levels of NF-kB, TNF-α, and iNOS. We then examined the effect of Neb and Met on LPS-induced changes in protein expression levels of NFkB, TNFα and iNOS. The LPS-treated cells exhibited elevated levels of NFkB, TNFα, and iNOS protein levels. Consistent with our RT-PCR data, cells treated with Neb showed a significant decrease in NFkB, TNFα and iNOS protein levels, while Met did not affect the expression (Figure 2D–F). Taken together, these data indicate that Neb but not Met alleviated the mRNA and protein levels of pro-inflammatory signaling molecules induced by LPS in H9c2 cells.

### 3.3. Effects of Neb and Met on LPS-Mediated Reduction in the Gene Associated with Mitochondrial Biogenesis

**Figure 3:**
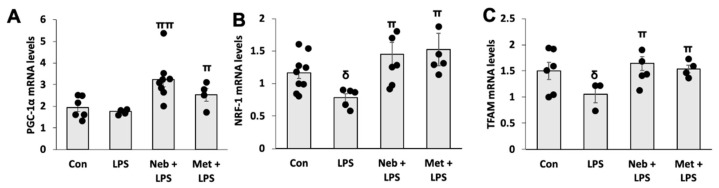
**Effects of Neb and Met on LPS-stimulated gene expression related to mitochondrial biogenesis in H9c2 cells.** mRNA levels of PGC-1α (**A**), NRF1 (**B**), and TFAM (**C**) were upregulated by both Neb and Met. ^δ^ *p* < 0.05 vs. Con, ^ππ^ *p* < 0.02, ^π^ *p* < 0.05 vs. LPS. Values are presented as means ± SEM N ≥ 6 for each treatment group.

Next, we examined the effects of LPS stimuli on molecular markers of mitochondrial biogenesis in the presence or absence of Neb or Met. Data from qPCR revealed that the treatment of cells with LPS decreased the mRNA expression of genes linked to mitochondrial biogenesis. The activation of upstream transcription factor PGC-1α co-activates the transcription of NRF-1, which binds and activates the TFAM promoter region that, in turn, upon translocation to mitochondria, increases mitochondrial DNA replication and gene transcription. We found that pretreatment with either Neb or Met prior to LPS stimulation increased the mRNA content of PGC-1α. Additionally, we further observed that treatment with LPS decreased the mRNA levels of nuclear respiratory-factor 1 (NRF1) (Figure 3B) and mitochondrial transcription-factor A (TFAM) (Figure 3C), which were significantly enhanced by pretreatments with either Neb or Met. However, no variations were observed in PGC-1α mRNA levels between untreated control and LPS, although LPS-treated cells revealed a decreasing trend. These data suggest that both Neb and Met promote mitochondrial biogenesis.

### 3.4. Effects of Neb and Met on Mitochondrial Dynamics and Mitochondrial ROS Production

**Figure 4:**
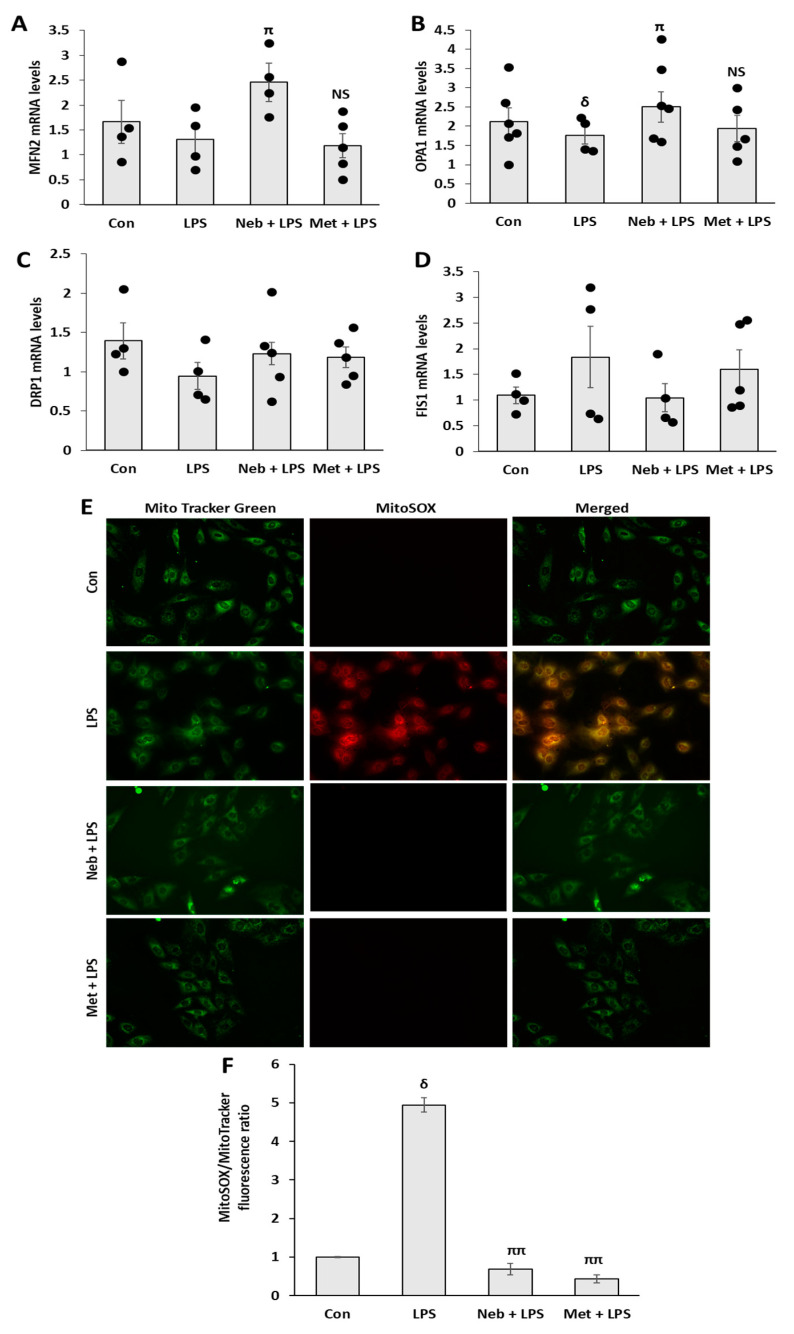
**Effects of Neb and Met on mitochondrial dynamics and generation of mitochondrial ROS in H9c2 cells.** Expression of genes associated with fusion MFN2 (**A**), OPA1 (**B**), and fission DRP1 (**C**) and FIS1 (**D**). (**E**) Representative images of H9c2 cells stained with MitoTracker Green and MitoSOX red. Increase in MitoSOX red fluoresence that co-localizes with MitoTracker Green, suggest an increase in mitochondrial ROS production in LPS treated cells. (**F**) Histogram showing the relative fluorescent intensity of MitoSOX red normalized to MitoTracker green. ^δ^ *p* < 0.05 vs. untreated (Con), ^ππ^ *p* < 0.02, ^π^ *p* < 0.05 vs. LPS and NS, nonsignificant vs. LPS. Values are presented as means ± SEM N ≥ 6 for each treatment group.

Mitochondrial dynamics (fission and fusion) are important for maintaining mitochondrial morphology. In response to oxidative stress induced by different stimuli, mitochondrial biogenesis and dynamics maintain the optimal energy output of mitochondria. Thus, we next assessed the impact of Neb and Met on LPS-stimulated mitochondrial fission- and fusion-related genes in H9c2 cells. Mitofusin 2 (MFN2), and optic atrophy 1 (OPA1) genes facilitate the fusion, whereas dynamin-related-protein 1 (DRP1) and fission-protein 1 (FIS1) promote fission. Treatment with Neb prior to LPS stimulation increased the expression of genes related to mitochondrial fusion, such as OPA-1 and MFN 2, compared to LPS alone, while the levels of DRP-1 and FIS1 remained unchanged (Figure 4A–D). In contrast to these findings, pretreatment with Met has no impact on genes related to fusion or fission. Additionally, LPS reduced OPA-1 gene levels compared to untreated control, while no substantial variations were seen in MFN 2 expressions between LPS and untreated control. Taken together, these findings demonstrate that Neb altered mitochondrial dynamics by increasing mitochondrial fusion in response to LPS. 

It is well known that increasing mitochondrial ROS production results in increased mitochondrial damage. Therefore, we investigated the effect of Neb and Met on LPS-induced mitochondrial ROS production in H9c2 cells using MitoSOX Red staining (Figure 4E,F). LPS clearly enhanced the fluorescent intensity as compared to control. A significant reduction in fluorescent intensity was observed after pretreatment with Neb and Met compared to LPS treatment. Collectively, these data reveal that pretreatment with Neb and Met suppresses LPS-induced mitochondrial ROS production in H9c2 cells.

### 3.5. Effects of Neb and Met on Genes Involved in Antioxidant Defense System

**Figure 5:**
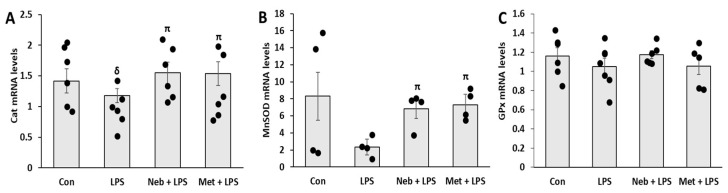
Effects of Neb and Met on antioxidant defense system in H9c2 cells. Expression of genes associated with cellular antioxidant defense system CAT (**A**), MnSOD (**B**) GPx (**C**). ^δ^ *p* < 0.05 vs. untreated (Con), ^π^ *p* < 0.05 vs. LPS.4.

To further delineate the protective effects of Neb and Met against LPS-induced oxidative stress, we assessed their effects on genes regulating the antioxidant defense system in response to LPS. A significant decrease was observed in catalase (CAT) mRNA levels by LPS treatment, whereas MnSOD showed a reducing trend. Pretreatment with either Neb or Met increased the expression of both CAT and MnSOD. On the other hand, no significant changes were seen in the mRNA levels of GPx in any treatment group (Figure 5A–C). Taken together, these data demonstrate that both Neb and Met stimulate the antioxidant defense system in response to LPS.

## 4. Discussion

In the current study, we compared the protective effects of β-AR blockers Neb and Met against LPS-induced injury in H9c2 cells. Our results demonstrate that both Neb and Met improve cell viability in the presence of LPS and reduce ROS production. Neb was potent in decreasing LPS-induced inflammation but not Met. Both drugs increased mitochondrial-related genes and Neb positively induces fusion-related genes, whereas Met did not change fusion or fission genes in LPS-treated H9c2 cells. 

The efficiency of β-blockers in reducing oxidative stress and inflammation in different models of cardiovascular diseases has been reported. For example, in a model of angiotensin II-induced cardiac dysfunction, treatment with Neb significantly reduced the oxidative stress expression of inflammatory and hypertrophic genes [23]. In a rodent model of obesity and insulin resistance, Neb improved myocardial remodeling, diastolic dysfunction and insulin metabolic signaling by inhibiting myocardial NADPH oxidase-mediated superoxide formation [11]. Additionally, Met decreased NADPH oxidase-2 (NOX2) in mice with intracerebral hemorrhage [13]. In patients with hypertension, only Neb reduced the oxidative stress-related biomarkers, but both Met and Neb reduced intercellular adhesion molecule-1 (ICAM-1), involved in the recruitment of circulating lymphocytes to the blood vessel wall. No changes in WBC, IL-6, high-sensitivity C-reactive protein (hsCRP), fibrinogen, or asymmetric dimethylarginine (ADMA) were observed with either Met or Neb, which may be explained by the relatively low cardiovascular risk of the participants [17]. In our study, we found that both Neb and Met were potent in reducing ROS production in cardiomyoblasts treated with LPS, but the changes in inflammatory markers were distinct between Neb and Met. 

In evaluating the effects of Neb compared to Met on correcting pulmonary arterial hypertension-related endothelial dysfunction, Neb was more potent than Met in decreasing inflammation [17]. In patients with pulmonary arterial hypertension, Neb but not Met decreased the expression of pro-inflammatory cytokines, such as IL-6 and MCP-1, in pulmonary endothelial cells in a dose-dependent manner. In addition, Neb dramatically decreased macrophage accumulation in the lung of an animal model of pulmonary hypertension, more than Met [10]. In our study, we also observed that Neb was more effective than Met in reducing pro-inflammatory gene expression in LPS-treated H9c2 cells. The reduced anti-inflammatory properties of Met could be explained by the antagonist effect of Met on vascular β2-AR [10]. In addition, the rise in NO bioavailability caused by Neb via the stimulation of AMPK/eNOS signaling is key to its anti-inflammatory and antioxidant properties in the presence of angiotensin II [24]. Nonetheless, suppression of sympathetic overactivity with Met, mitigates cardiac dysfunction and reduces cardiac inflammation in a mouse model of intracerebral hemorrhage (ICH). The expression of the proinflammatory mediator’s TNF-α and IL-6 were shown to be reduced in the Met-treated ICH mice compared with saline-treated ICH mice. Met also led to reduced NF-κB pathway activation, MCP-1 expression, and macrophage and neutrophil infiltration in the heart post-ICH [13]. 

Mitochondrial dysfunction plays a critical role in cardiac dysfunction [25,26]. We previously reported the beneficial effects of Neb against mitochondrial dysfunction in angiotensin II-induced pathology in H9c2 cardiomyoblasts. Neb induces the expression of genes associated with mitochondrial biogenesis and dynamics in the presence of angiotensin II [24]. The exposure of 3T3-L1 adipocytes to Neb for 24 h increased mitochondrial synthesis and metabolism primarily via mechanisms related to eNOS/cGMP pathway and β3-AR receptor activation [6]. Additionally, Neb treatment restored the myocardial mitochondrial number in Zucker obese rats to levels similar to those in the Zucker lean rats and improved mitochondrial sarcomere organization [11]. In contrast, the short duration of Met treatment did not improve mitochondrial function in the hypertrophied right ventricle of pulmonary hypertensive rats [27]. Although β-blockers have demonstrated their efficacy in reducing mortality during myocardial infarction and chronic heart failure, the use of beta-blockers during sepsis has both favorable and unfavorable effects on cardiac function. It seems that β-blockers can regulate the increased heart rate that is frequently observed in patients with sepsis. Thus, β-blockade during sepsis has the potential to enhance myocardial oxygen demand and myocardial efficiency, which, in turn, attenuates the incidence of arrhythmias and alleviates cardiac workload [28,29]. However, the administration of β-blockers has the potential to decrease blood pressure. This could worsen the pre-existing hypotension, a prevalent complication observed in sepsis, and consequently compromise tissue perfusion, which is a matter of considerable significance [18]. In our study, Neb and Met were effective in inhibiting mitochondrial ROS production and the induction of mitochondrial biogenesis-related genes; however, Neb was more potent than Met in increasing the fusion-related genes. These data indicate that Met does not affect mitochondrial dynamics in the presence of LPS in cardiomyoblasts.

In conclusion, the data presented here for the first time revealed that Neb and Met treatment significantly ameliorate LPS-induced injury and oxidative stress. Additionally, Neb but not Met reduced the LPS-induced expression of proinflammatory genes in H9c2 cardiomyoblasts. Treatment with both Neb and Met improved the expression of genes linked with mitochondrial biogenesis. However, the expression of genes related to mitochondrial fusion was upregulated by Neb but not Met. Taken together, these findings demonstrate that Neb but not Met downregulates LPS-induced proinflammatory factors by enhancing mitochondrial biogenesis and fusion. These data, for the first time, demonstrate the comparative effects of Neb and Met on LPS-induced injury in H9c2 cardiomyoblasts. However, animal studies are still needed to evaluate the physiological impacts of these drugs on LPS-induced cardiac injury. Evaluating the inflammation of cardiac muscle, mitochondrial changes, and cardiac function would be important in these mice. Additionally, human relevance data are lacking in our study. Having evidence from both humans and animals would enhance the clinical interventions using b-blockers in cardiac diseases.

## Figures and Tables

**Table 1:** Primer sequences.

Gene	Forward Primer	Reverse Primer	Accession Number
TNF-α	CACTCAGGCATCGACATTCG	CACCGGCAAGGATTCCAA	XM_032888689
iNOS	CGGCCACCAGCTTCTTCA	TGCTTACAGGTCTACGTTCAAGACAT	XM_032912147
NF-kB	TGAGTCCCGCCCCTTCTAA	TGATGGTCCCCCCAGAGA	NM_00127671
PGC-1α	ACTCAGCAAGTCCTCAGTGC	TTCTGGTGCTGCAAGGAGAG	NM_031347
TFAM	TGTCATTGGGATTGGGCACA	AGATGCACGCACAGTCTTGA	XM_032888687
NRF1	CATGGCCCTTAACAGTGAAGC	TGGTCCATGCATGAACTCCA	NM_001100708
MFN2	TGTTCAGAGGCCATCGGTTC	TCCACCTGTCTGAACTTCACC	XM_008764288
FIS1	GGGTTACATGGATGCCCAGA	TTTGGGCAACAGCTCCTCC	XM_032886584
DRP1	ACAACAGGAGAAGAAAATGGAGTTG	TGGATTGGCTCAGGGCTTAC	NM_053655
OPA1	TCTTCACTGCGGGTACACCT	TCCTTCTCCAAACGCTCCAG	XM_017597866
MnSOD	ACCACAGGCCTTATTCCACT	TACAACAGCTCAGCCACAGT	Y00497
CAT	TCCCAGAAGCCTAAGAATGCA	GCGATGATTACTGGTGAGGCT	NM_012520
GPx	CAGTCCACCGTGTATGCCTT	TGCCATTCTCCTGATGTCCG	NM_030826
β-actin	CAACGTCACACTTCATGATGGA	ATGCCCCGAGGCTCTCTT	XM_032887061

## Data Availability

All relevant data are within the manuscript and its Supporting file.

## Supplementary Materials

The following supporting information can be downloaded at: https://www.mdpi.com/article/10.3390/cimb45110583/s1. Figure S1: Cell viability by crystal violet assay following treatment with Neb and Met. ^δ^ *p* < 0.02, vs. untreated (Con), ^ππ^ *p* < 0.02 vs. LPS. Values are presented as means ± SEM N ≥ 6 for each treatment group.
