# Supplementary material for: Comparison of the Protective Effects of Nebivolol and Metoprolol against LPS-Induced Injury in H9c2 Cardiomyoblasts
Source: Curr Issues Mol Biol. 2023 Nov 20;45(11):9316–27. doi: 10.3390/cimb45110583 (PMC10670410; doi:10.3390/cimb45110583)
Supplement: Supplementary file 1 [file cimb-45-00583-s001.zip › cimb-2669729-supplementary.pdf]

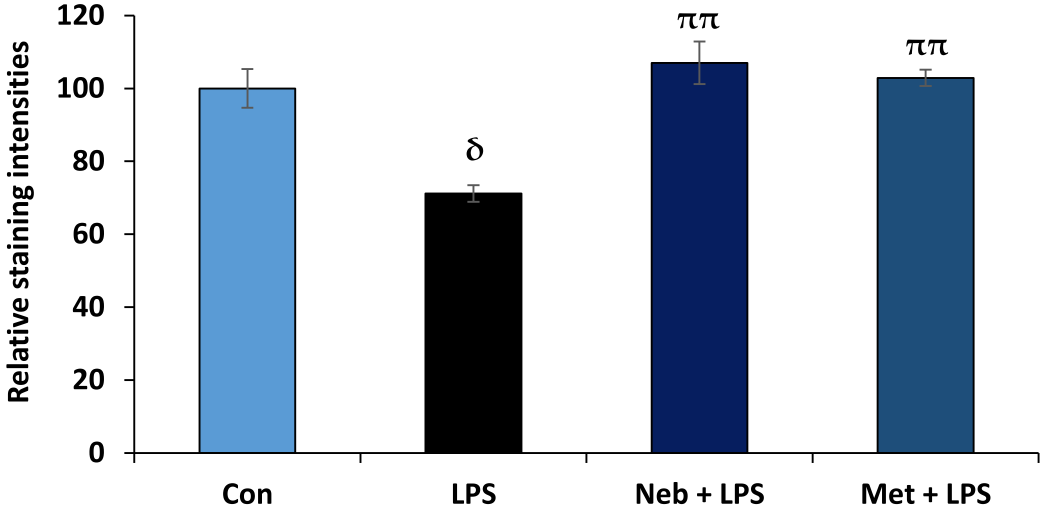

Supplementary Figure S1: Cell viability by crystal violet assay following treatment with Neb and Met. δ $p < 0.02$, vs. untreated (Con), ππ $p < 0.02$ vs. LPS. Values are presented as means ± SEM N ≥ 6 for each treatment group.
